# 

**Published:** 1924-08

**Authors:** 


### RECENT APPOINTMENTS.

### Medical Officers, &c.

Campbell, Helen M.B., Assistant Medical Officer, Warrington.

Champneys, Weldon, B.Ch., Assistant Medical Officer, Willesden.

Gray, Alice, M.A., M.B., Resident Medical Officer, Municipal Hospital, Willesden.

Moir, P. J., M.C., M.B., Ch.B., F.R.C.S.; Honorary Assistant Surgeon, Leeds General Infirmary.

Rees, John Owen Morgan, House Surgeon, The Guest Hospital, Dudley; Assistant Medical Officer, Mid-Wales Counties Mental Hospital.

Sullivan, Annie B., M.B., School Medical Officer, Cork.

### Public Health and Hospital Appointments.

Adams, L. A., C.M.B.; Matron, Poor Law Institution, Hull.

Anger, Lilian E. B., Health Visitor, St. Pancras; School Nurse, Finchley.

Bennett, Isabella E.; Superintendent, Queen Victoria Jubilee Institute, Harrogate.

Boyd, Margaret; Superintendent (Queen Victoria Jubilee Nurses' Institute), Aberdeen Training Home.

Carden, Beryl; Superintendent, South Islington Welfare Centre.

Cassidy, Mary Bridget; Health Visitor, Bournemouth.

Clark, G. A., M.D.; Lecturer in Physiology, Sheffield University.

Courts, H. L.; Matron, Hertfordshire Maternity Home, Hitchin.

Davies, Elizabeth Watkin; Health Visitor and School Nurse, Cardiganshire.

Donaldson, Mary; Matron, Stockton-on-Tees Fever Hospital.

Evans, J. H.; Borough and Port Sanitary Inspector, Boston.

Fairbairns, Maude; School Nurse, Wood Green, N.

Folliott, Lucy M., Addenbrooke's Hospital, Cambridge; Matron, North Cambs Hospital,

Gaskin, E. L.; Matron, Central London Throat, Nose and Ear Hospital.

Green, Matilda; Health Visitor, Barnsley.

Hewlett, Professor R. T.; Examiner for Diploma in Public Health.

Husband, Margaret, C.M.B.; Assistant Matron, Royal Infirmary, Cardiff.

Jones, Lily; School Nurse, Walsall.

Lowe, Eveline; School Nurse, Leeds.

Mortimer, Jane; Health Visitor, Preston.

Savery, Marguerite, Matron, Isolation Hospital, Dover; Matron, Worcester Infirmary.

Stevens, F. J., M.B., Medical Officer for Camberwell; Examiner for Diploma in Public Health.

Thompson, E., C.M.B.; Matron, West Suffolk Hospital Bury St. Edmunds.

Turner, Mary A.; Matron, Wrexham Infirmary.

Willey, Dorcas A.; Matron, Whitby Hospital.

Willis, E. M., Matron, Royal Chest Hospital; Matron, Royal Northern Hospital, Holloway.

Wood, Annie; School Nurse, Halifax.

### ANSWERS TO CORRESPONDENTS.

J. J.—The bedding and blankets must be really good. Messrs. D. Stewart & Co., of 16, Staining Lane, Gresham-stree, E.C. 2, the well-known makers of hospital bedding and blankets might help you.

Jane.—Your invalid sister could spend her convalescence in healthy, pleasant surroundings, and for a very moderate charge at: Convalescent Home, Otham, Maidstone; St. Mary's and St. Saviour's Convalescent Home, Queen's Road, Brighton; Village Home, Findon, Worthing; St. Joseph's Convalescent Home, Bournemouth; or All Saints' Convalescent Hospital, Eastbourne.

Teacher.—You probably mean the Professional and Business Women's Hospital League. The secretary is Miss Ransom, 197, Edgware Road, W. 2.

### Managerial Notices.

*All letters on Business matters must be addressed to the Manager, "The Hospital and Health Review," 28 and 29 Southampton Street, London, W.C. 2.*

Rates for Subscription (payable in advance).

| | | |
|---|---|---|
| Three Months (including Postage) | .. .. | 1s. 9d. |
| Six Months do. | .. .. | 3s. 6d. |
| Twelve Months do. | .. .. | 7s. 0d. |

**It is especially requested that remittances be made by Cheque or Postal Order and not Stamps.**

Subscriptions, which may commence at any time, are payable in advance. Cheques and Post-Office Orders should be crossed Westminster Bank, Covent Garden Branch, and made payable to the Manager of The Hospital and Health Review.

***Advertisements.*** Scale of charges and particulars of special positions may be obtained on application to the Manager.

*SUBSCRIPTION FORM.*

*Please send me* The Hospital and Health Review *for.............. months, commencing with your issue of ................, for which please find enclosed ........*

*Name* ..............................

*Address* ..............................

..............................

*Date* ..............................

*To*.............................*Newsagent*

